# Optimal Fractional-Order Active Disturbance Rejection Controller Design for PMSM Speed Servo System

**DOI:** 10.3390/e23030262

**Published:** 2021-02-24

**Authors:** Pengchong Chen, Ying Luo, Yibing Peng, Yangquan Chen

**Affiliations:** 1 School of Mechanical Science and Engineering, Huazhong University of Science and Technology, Wuhan 430070, China; pc_chen@hust.edu.cn; 2 School of Engineering, University of California, 5200 N.Lake Road, Merced, CA 95343, USA; ychen53@ucmerced.edu

**Keywords:** PMSM speed servo, ADRC design, fractional-order control, frequency-domain specifications, time-domain performance

## Abstract

In this paper, a fractional-order active disturbance rejection controller (FOADRC), combining a fractional-order proportional derivative (FOPD) controller and an extended state observer (ESO), is proposed for a permanent magnet synchronous motor (PMSM) speed servo system. The global stable region in the parameter (*K_p_*, *K_d_*, μ)-space corresponding to the observer bandwidth *ω_o_* can be obtained by D-decomposition method. To achieve a satisfied tracking and anti-load disturbance performance, an optimal ADRC tuning strategy is proposed. This tuning strategy is applicable to both FOADRC and integer-order active disturbance rejection controller (IOADRC). The tuning method not only meets user-specified frequency-domain indicators but also achieves a time-domain performance index. Simulation and experimental results demonstrate that the proposed FOADRC achieves better speed tracking, and more robustness to external disturbance performances than traditional IOADRC and typical Proportional-Integral-Derivative (PID) controller. For example, the *J_ITAE_* for speed tracking of the designed FOADRC are less than 52.59% and 55.36% of the *J_ITAE_* of IOADRC and PID controller, respectively. Besides, the *J_ITAE_* for anti-load disturbance of the designed FOADRC are less than 17.11% and 52.50% of the *J_ITAE_* of IOADRC and PID controller, respectively.

## 1. Introduction

Permanent magnet synchronous motor (PMSM) is mostly accepted in motion control applications due to its advantages, such as compact structure, high power density, high air-gap flux density, and high efficiency [1]. Currently, PMSM is widely applied in industries. Proportional-Integral- Derivative (PID) controller can get reasonable transient and steady-state response with three adjustable parameters. However, the satisfied tracking and anti-load disturbance performances are difficult to be obtained simultaneously [2].

Active disturbance rejection control (ADRC), proposed by Prof. Jingqing Han, is a solution to the problem of internal and external disturbances raised by Tsien and Horowitz [3,4,5]. The core of ADRC is the extended state observer (ESO), which treats the external disturbances and internal uncertainties as the total disturbance and rejects them actively. However, due to the complexity of the structure of the ADRC and the difficulty of tuning parameters, its applications in the industrial fields were still limited until the linear ADRC (LADRC) proposed in Reference [6]. The research and application of ADRC have increased significantly in recent years and have proved that ADRC can achieve satisfy anti-load performance [7,8,9,10]. To simplify the tuning parameters of ADRC, the observer bandwidth $\omega_{o}$ and controller bandwidth $\omega_{c}$ are the only tuning parameters, which is the basis of most of the studies. For example, Caifen Fu proposed a generalized ADRC (GADRC) method with known plant information and a tuning method to enhance control performance [11]. Xiangyang Zhou proposed a parameter tuning strategy using the genetic algorithm (GA) for ADRC to increase the stabilization accuracy and robustness to disturbance [12]. However, this method is only approximate, and its application is limited by the complexity of the plant. Thus, to achieve a better control performance in terms of tracking and anti-load disturbance and be widely used in the PMSM field, a fractional order ADRC based on fractional-order proportional derivative (FOPD) controller and the design method based on frequency-domain are proposed.

Since fractional-order calculus was created in 1695 [13,14], there has been a great development as a purely theoretical subject of mathematics. Recently, the utilization of fractional-order calculus continues to increase, not only in mathematics but also in biology, physics, electromagnetic, engineering, and other areas of science [15,16,17,18,19]. Particularly in the field of control engineering, various forms of fractional-order (FO) controllers have been demonstrated that can achieve better control performance, such as the fractional-order proportional-integral (FOPI) controller, FOPD controller, fractional-order (proportional-derivative) (FO(PD)) controller, fractional-order proportional-integral-derivative (FOPID) controller, and so on [20,21,22,23,24]. The tuning methods for FO-type controllers mainly include minimizing a performance index in time-domain [25,26] and satisfying pre-specified frequency-domain specifications [22,27]. The popular time-domain indexes and frequency-domain specifications are the integral square error (ISE), the integral time absolute error (ITAE), phase margin, gain margin, and gain crossover frequency.

Due to the fact that, fractional-order PD controller has been proven to achieve better tracking performance with less overshoot and faster response than traditional integer-order PD controller [28,29]. Fractional active disturbance rejection control (FADRC) combined with fractional-order ESO (FOESO) and fractional-order proportional derivative (FOPD) controller was proposed by Reference [30] to enhance the control performance for a fractional-order system (FOS), where the FADRC stability and frequency-domain characteristics were analyzed. However, the proposed FADRC strictly constraints the orders of FOPD and FOESO corresponding to the order of the FOS. A fractional-order active disturbance rejection control (FOADRC) strategy including a nonlinear ESO to achieve precise trajectory tracking performance for a newly designed linear motor was presented in Reference [31]. However, the parameters tuning rule was not presented in References [30,31]. Pengchong Chen [32] proposed a fractional-order ADRC strategy combined with the FOESO and a simple proportional controller. The tuning approach based on frequency-domain specifications was presented for the proposed fractional-order ADRC and traditional integer-order ADRC. However, the analytical design method based on frequency-domain specifications for ADRC-type has not been studied, which is crucial for industrial applications.

In this paper, aiming to realize satisfied tracking and anti-load disturbance performance for the PMSM speed servo system, a FOADRC combining fractional-order proportional derivative (FOPD) controller and ESO is proposed. The total disturbances are estimated and compensated by the ESO; the FOPD controller achieves optimal tracking performance. A FOADRC design strategy is proposed for satisfying user-specified frequency-domain indexes and achieving a time-domain performance index. The main contributions in this paper can be stated as follows: (1) A FOADRC structure for the PMSM speed servo system is proposed combining a FOPD controller and an ESO. The stability boundaries for controller parameters have been clearly analyzed. (2) An optimal FOADRC design scheme for satisfying time-domain and frequency-domain specifications simultaneously is proposed, which is also applicable to IOADRC. (3) PMSM speed servo simulation and experimental results are presented to show the control performance advantages of the designed optimal FOADRC over the traditional IOADRC and the typical PID controller.

The next sections of the paper are organized as follows: Section 2 gives the background of fractional-order calculus and the PMSM speed servo system. The structure of FOADRC for the PMSM speed servo system is proposed. In Section 3, the stability boundary analysis of FOADRC is given. The design scheme is presented with an example in Section 4. In Section 5 and Section 6, simulation and experimental results are presented to demonstrate the performance of the PMSM servo system using the designed optimal FOADRC. The conclusion is indicated in Section 7.

## 2. Background and Preliminaries

### 2.1. The Tuning Methods for ADRC

In this subsection, some of the existing methods are summarized chronologically: In Reference [33], an auto-tuning method for $\omega_{o}$ and $\omega_{c}$ based on noise level in control signal was proposed. Behzad et al. [34] proposed a systematic procedure for tuning ADRC parameter based on the desired settling time. A tuning procedure was proposed in Reference [10] for modified ADRC by systematically varying $b_{0}$, $\omega_{c}$, and $\omega_{o}$. All the above methods have the element of trial and error, although they are effective. The operator should have a thorough understanding; otherwise, it is difficult to apply in practice. To solve this issue, the quantitative tuning rule based on frequency-domain specifications was proposed in Reference [35] to satisfy the phase and gain margin for first-order plus time delay system was proposed. However, this method is based on the FOPTD systems and only applicable for lag-dominated plants.

### 2.2. Fractional-Order Calculus

Fractional-order calculus means generalizing integral and differential operators to the fractional operators. The continuous integral-differential operator is defined as follows:

(1) t0Dtα≜dαdtα,Re(α)>0;1,Re(α)=0;∫t0t(dτ)−α,Re(α)<0;

where $t_{0}$ and *t* are the start time and the end time of the integration, respectively. The term α is the fractional-order. Re(α) is the real part of α.

In this paper, the following Caputo definition of fractional derivative is utilized to realize the FOPD controller [36],

(2) $${}_{0}D_{t}^{\alpha }f\left(t\right)=\frac{1}{\Gamma (n-\alpha )}\int_{t_{0}}^{t}\frac{f^{\left(n\right)}\left(\tau \right)d\tau }{{\left(t-\tau \right)}^{\alpha -n+1}},$$

where *n* is an integer which satisfied the case n−1<α<n, $f^{\left(n\right)}\left(\tau \right)$ is the *n*th derivative of the f(τ), and the Γ(x) is the Gamma function with the definition,

(3) $$\Gamma \left(x\right)=\int_{0}^{\infty }t^{x-1}e^{-t}dt.$$

The Laplace transform of the Caputo derivative is:

(4) $$L{}_{0}D_{t}^{\alpha }f\left(t\right);s=s^{\alpha }F\left(s\right)-\sum_{k=0}^{n-1}s^{\alpha -k-1}f^{k}\left(0\right)\;\;n-1<\alpha \leq n$$

### 2.3. Problem Formulation

**Figure 1:**
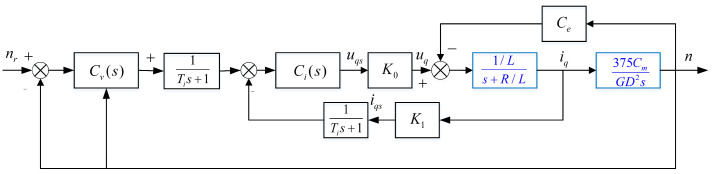
The permanent magnet synchronous motor (PMSM) speed closed-loop control system.

**Figure 2:**
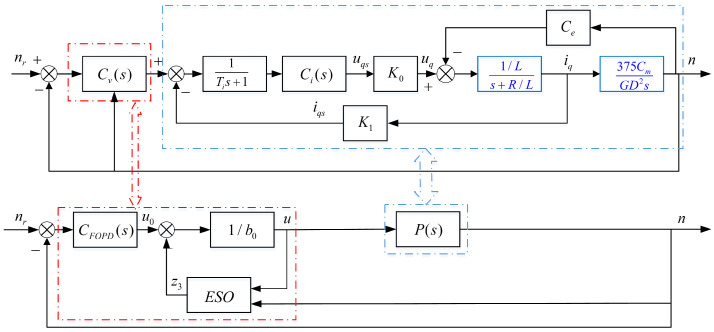
The PMSM speed closed-loop control system with fractional-order active disturbance rejection control (FOADRC).

The PMSM speed closed-loop control system is shown in Figure 1, which can be equivalent to Figure 2.

Where $n_{r}$ and *n* are the reference speed and actual speed, respectively, and the unit is rpm, $i_{q}$ and $u_{q}$ are the *q*-axis current and voltage, respectively, $C_{e}$ is the induced electromotive force coefficient, $K_{0}$ and $K_{1}$ are the voltage and current conversion factors, respectively, $T_{i}$ is the filter time constant, *R* and *L* are the stator equivalent resistance and inductance, respectively, $C_{m}$ is the torque constant, $GD^{2}$ is the flywheel inertia, and the unit is N·m${}^{2}$; $u_{0}$ is the output of the FOPD controller, $z_{3}$ is the output of the ESO, and *u* is the control law.

Assume that hysteresis and eddy current loss, saturation nonlinear factor of magnetization curve and friction are ignored, and there is no damper winding on the rotor. The transfer functions of the electromagnetic and mechanical parts, which are needed to be identified, are shown with zero initial conditions,

(5) $$\begin{array}{cc} & G_{1}\left(s\right)=\frac{1/L}{s+R/L}, \end{array}$$

(6) $$\begin{array}{cc} & G_{2}\left(s\right)=\frac{375C_{m}}{GD^{2}s}. \end{array}$$

A nonlinear identification method based on output-error is adopted to obtain the model parameters [37,38]. Thus, the electromagnetic and mechanical models can be identified as:

(7) $$\begin{array}{c} G_{1}\left(s\right)=\frac{154.8568}{s+446.188}, \end{array}$$

(8) $$\begin{array}{c} G_{2}\left(s\right)=\frac{367.6516}{s}. \end{array}$$

**Figure 3:**
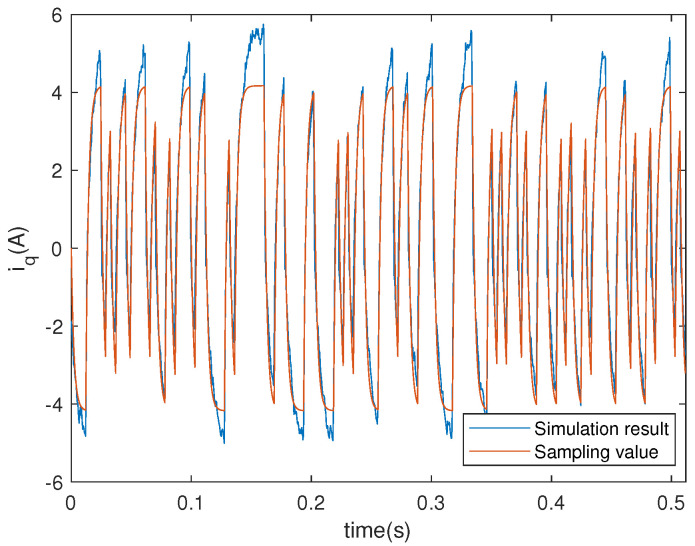
Actual system output and model output of the electromagnetic part.

**Figure 4:**
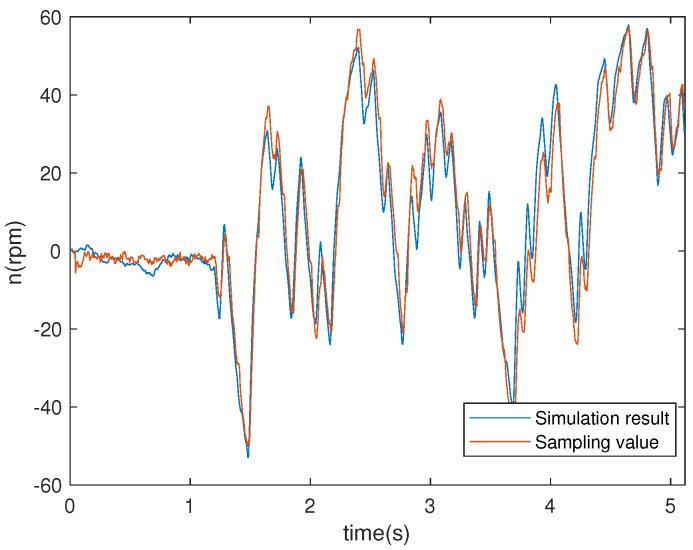
Actual system output and model output of the mechanical part.

The comparison between simulation results based on the obtained models of the electromagnetic and mechanical parts and experimental results are presented in Figure 3 and Figure 4. For more details on the system identification scheme, the reader may refer to Reference [38].

A PI controller with $T_{i}$ is applied to control the current-loop system,

(9) $$C_{i}\left(s\right)=K_{pi}\left(1+\frac{1}{T_{i}s}\right).$$

Based on the above identification results, the speed servo plant of PMSM can be obtained:

(10) $$P\left(s\right)=\frac{Y(s)}{U(s)}=\frac{b}{s(s+a)},$$

where a=26.08, b=383.635, *U* is the input reference current, and *Y* is the actual speed.

### 2.4. The Structure of the FOADRC

In this paper, a FOADRC with a FOPD controller is proposed. The structure of the FOADRC is shown in Figure 2.

The load disturbance *d* is considered when the PMSM is running. Thus, Equation (10) can be rewritten as (11), considering the external disturbance *d*:

(11) $$\ddot{y}=bu-a\dot{y}+d=bu+f,$$

where *u* and *y* are the input and output, respectively, *d* is the external disturbance, and *f* is the total disturbance, which contains the internal dynamics and external disturbance *d*.

The FOADRC includes a FOPD controller and an ESO. The ESO of the FOADRC is used to estimate the total disturbance *f*. Suppose that *f* is differentiable, $h=\dot{f}$, then the state space form of (11) is:

(12) $$\left\{\begin{array}{c} \dot{x}=Ax+Bu+Eh,\\ y=Cx, \end{array}\right.$$

where

$$\left\{\begin{array}{c} A=\left[\begin{array}{ccc} 0 & 1 & 0\\ 0 & 0 & 1\\ 0 & 0 & 0 \end{array}\right],B=\left[\begin{array}{c} 0\\ b\\ 0 \end{array}\right],E=\left[\begin{array}{c} 0\\ 0\\ 1 \end{array}\right],\\ C=\left[\begin{array}{ccc} 1 & 0 & 0 \end{array}\right],x=\left[\begin{array}{c} x_{1}\\ x_{2}\\ x_{3} \end{array}\right]. \end{array}\right.$$

Then, the ESO is designed for (12),

(13) $$\left\{\begin{array}{c} \dot{z}=Az+Bu+L\left(y-\hat{y}\right),\\ \hat{y}=Cz, \end{array}\right.$$

where $L={\left[\beta_{1}\;\beta_{2}\;\beta_{3}\right]}^{T}$ is the gain of the ESO; $z={\left[z_{1}\;z_{2}\;z_{3}\right]}^{T}$, $z_{1}$, $z_{2}$, and $z_{3}$ are the outputs of the ESO: $z_{1}$ and $z_{2}$ will estimate *y* and its derivative, and $z_{3}$ will estimate total disturbance *f*. The estimated total disturbance will be rejected as

(14) $$u=\frac{u_{0}-z_{3}}{b},$$

where $u_{0}$ is the output of the FOPD controller.

The FOPD controller is:

(15) $$C_{FOPD}\left(s\right)=K_{p}+K_{d}s^{\mu },$$

where $K_{p}$ and $K_{d}$ are the proportional and derivative gains, and μ∈(0,2) is the derivative order.

## 3. Stability Boundary Analysis

Because the stability is the minimal requirement for the control system, it is desirable to determine the complete stabilizing FOPD parameters before controller design.

From (13), with Laplace transform,

(16) $$Z_{3}\left(s\right)=\frac{\beta_{3}s^{2}}{s^{3}+\beta_{1}s^{2}+\beta_{2}s+\beta_{3}}Y\left(s\right)-\frac{b\beta_{3}}{s^{3}+\beta_{1}s^{2}+\beta_{2}s+\beta_{3}}U\left(s\right).$$

where

(17) $$\beta_{1}=3\omega_{o},\;\beta_{2}=3\omega_{o}^{2},\;\beta_{3}=\omega_{o}^{3}.$$

In this way, all three of the observer poles will be placed at $-\omega_{o}$, which is the observer bandwidth [6].

According to (10), (14) and (16), the plant with disturbance compensation is

(18) $$P_{c}\left(s\right)=\frac{Y(s)}{U_{0}\left(s\right)}=\frac{s^{3}+\beta_{1}s^{2}+\beta_{2}s+\beta_{3}}{s^{5}+\left(a+\beta_{1}\right)s^{4}+\left(a\beta_{1}+\beta_{2}\right)s^{3}+\left(a\beta_{2}+\beta_{3}\right)s^{2}},$$

where $U_{0}\left(s\right)$ is the Laplace transform of $u_{0}$.

**Figure 5:**
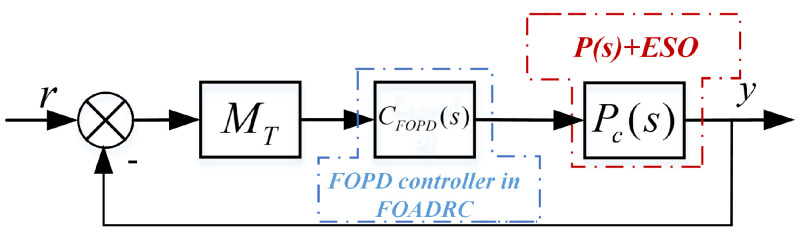
The PMSM speed closed-loop control system based on FOADRC.

In the considered feedback control system as shown in Figure 5, $P_{c}\left(s\right)$ is the controlled plant, and $C_{FOPD}\left(s\right)$ is the designed FOPD controller. There are four tuning parameters: $K_{p}$, $K_{d}$, μ, and $\omega_{o}$.

In Figure 5, $M_{T}$ is a Gain-Phase Margin Tester,

(19) $$M_{T}\left(A,\phi \right)=Ae^{-j\phi },$$

where *A* and ϕ are the gain margin and phase margin, respectively.

The closed-loop transfer function of the control system in Figure 5 can be obtained as:

(20) $$G\left(s\right)=\frac{M_{T}\left(A,\phi \right)C_{FOPD}\left(s\right)P_{c}\left(s\right)}{1+M_{T}\left(A,\phi \right)C_{FOPD}\left(s\right)P_{c}\left(s\right)}.$$

Hence, the characteristic equation of G(s) is:

(21) $$\begin{array}{c} D\left(K_{p},K_{d},\mu ,A,\phi ;s\right)=(s^{5}+\left(a+\beta_{1}\right)s^{4}+\left(a\beta_{1}+\beta_{2}\right)s^{3}+\\ \left(a\beta_{2}+\beta_{3}\right)s^{2})+Ae^{-j\phi }\left(K_{p}+K_{d}s^{\mu }\right)\left(s^{3}+\beta_{1}s^{2}+\beta_{2}s+\beta_{3}\right). \end{array}$$

The range of the fractional-order μ is defined as μ∈(0,2). The lower limit of $\omega_{o}$ depends on the pre-specified gain crossover frequency $\omega_{gc}$, and the upper limit of $\omega_{o}$ is set to 800 rad/s in this paper. The boundaries of the stable region which are determined by real root boundary (RRB), infinite root boundary (IRB) and complex root boundary (CRB) can be obtained by the D-decomposition method [39,40].

- Real root boundary (RRB): The RRB is defined by the equation $D(K_{p},K_{d},\mu ,A,\phi ,\omega_{o};s=0)=0$, so the boundary of $K_{p}$ is:
  (22) $$K_{p}=0.$$
- Infinite root boundary (IRB): Due to the relative order of $P_{c}\left(s\right)$ is 2, $K_{d}$ has no boundary restrictions.
- Complex root boundary (CRB): Putting jω for *s* into (21), the CRB can be described by $D(K_{p},K_{d},\mu ,A,\phi ,\omega_{o};s=j\omega )=0$, yielding
  (23) $$\begin{array}{c} D\left(K_{p},K_{d},\mu ,A,\phi ,\omega_{o};j\omega \right)=({\left(j\omega \right)}^{5}+\left(a+\beta_{1}\right){\left(j\omega \right)}^{4}\\ +\left(a\beta_{1}+\beta_{2}\right){\left(j\omega \right)}^{3}+\left(a\beta_{2}+\beta_{3}\right){\left(j\omega \right)}^{2})\\ +Ae^{-j\phi }\left(K_{p}+K_{d}{\left(j\omega \right)}^{\mu }\right){(\left(j\omega \right)}^{3}+\\ \beta_{1}{\left(j\omega \right)}^{2}+\beta_{2}\left(j\omega \right)+\beta_{3})=0. \end{array}$$

The real part and the imaginary part of (23) can be obtained,

(24) $$\begin{array}{cc} & F_{1}+A\left(K_{p}E_{5}+K_{d}E_{6}\right)=0, \end{array}$$

(25) $$\begin{array}{cc} & F_{2}+A\left(K_{p}E_{7}+K_{d}E_{8}\right)=0, \end{array}$$

where

$$\begin{array}{cc} & F_{1}=\left(a+\beta_{1}\right)\omega^{4}-\left(a\beta_{2}+\beta_{3}\right)\omega^{2},\\ & F_{2}=\omega^{5}-\left(a\beta_{1}+\beta_{2}\right)\omega^{3},\\ & E_{3}=-\omega^{3}+\beta_{2}\omega ,\;E_{4}=-\beta_{1}\omega^{2}+\beta_{3},\\ & E_{5}=cos\left(\phi \right)E_{4}+sin\left(\phi \right)E_{3},\\ & E_{6}=\omega^{\mu }cos\left(\frac{\mu \phi }{2}-\phi \right)E_{4}-\omega^{\mu }sin\left(\frac{\mu \pi }{2}-\phi \right)E_{3},\\ & E_{7}=cos\left(\phi \right)E_{3}-sin\left(\phi \right)E_{4},\\ & E_{8}=\omega^{\mu }cos\left(\frac{\mu \phi }{2}-\phi \right)E_{3}-\omega^{\mu }sin\left(\frac{\mu \pi }{2}-\phi \right)E_{4}. \end{array}$$

From (24) and (25),

(26) $$\begin{array}{cc} & K_{d}=\frac{F_{2}E_{5}-F_{1}E_{7}}{A(E_{6}E_{7}-E_{5}E_{8})}, \end{array}$$

(27) $$\begin{array}{cc} & K_{p}=-\frac{F_{2}+AK_{d}E_{8}}{E_{7}}. \end{array}$$

So, given a fixed fractional-order μ and a fixed $\omega_{o}$, the stable and unstable regions in the parameter plane can be separated according to the RRB and CRB with A=1, $\phi =0^{\circ }$.

**Figure 6:**
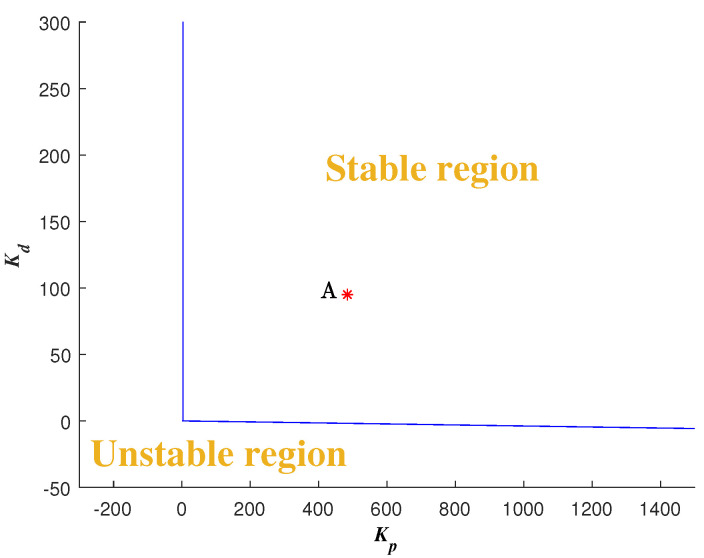
Stable region with μ=0.9, $\omega_{o}=200\;\mathrm{rad}/s$ and the designed $K_{p}$ and $K_{d}$ with $\phi_{m}=80^{\circ }$, $\omega_{gc}=50\;\mathrm{rad}/s$.

**Figure 7:**
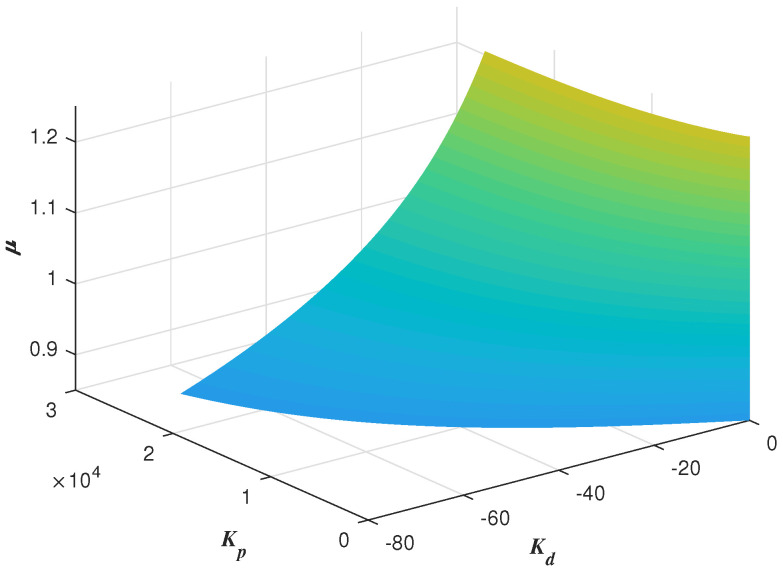
Global stable region with $\omega_{o}=200\;\mathrm{rad}/s$.

One example is given as: Choosing μ=0.9 and $\omega_{o}=200\;\mathrm{rad}/s$, draw the curve of $K_{p}$ w.r.t. $K_{d}$ and the line $K_{p}=0$ according to CRB and RRB, respectively. Detect the stable region by testing a random point [41] as shown in Figure 6. Sweeping all the μ∈(0,2), the global stable region can be obtained as shown in Figure 7. Thus, with different $\omega_{o}$, different global stable regions can be determined.

## 4. FOADRC/IOADRC Design Strategy

### 4.1. Controller Design Specifications

In this paper, two frequency-domain specifications, gain crossover frequency ($\omega_{gc}$) and phase margin (PM), are applied to tune FOADRC:

- Gain crossover frequency
  (28) $$|C_{FOPD}\left(j\omega_{gc}\right)P_{c}\left(j\omega_{gc}\right){|}_{dB}=0.$$
- Phase margin
  (29) $$\arg[C_{FOPD}\left(j\omega_{gc}\right)P_{c}\left(j\omega_{gc}\right)]=-\pi +PM.$$

To achieve an optimal dynamic control performance, a time-domain specification, ITAE, is also applied for the FOADRC design:

- ITAE
  (30) $$J_{ITAE}=\int_{0}^{\infty }\left|e\left(t\right)\right|t\;dt,$$
  where e(t) is the deviation between the reference input and the actual output.

### 4.2. The Optimal FOADRC Design for PMSM Speed Servo Plant

#### 4.2.1. The FOADRC Satisfying the Frequency-Domain Specifications

The Gain-Phase Margin Tester can provide information for satisfying the given gain margin or phase margin [40]. Setting A=1 and $\phi =\phi_{m}$, plot the curve according to Equations (26) and (27) with ω increasing from 0. Every point on the above curve fulfills the given phase margin $\phi_{m}$.

The characteristic equation of Equation (20) is,

(31) $$1+M_{T}\left(A,\phi \right)C_{FOPD}\left(s\right)P_{c}{\left(s\right)|}_{s=j\omega }=0,$$

which denotes the open-loop transfer function T(s) equals to −1,

(32) $$T{\left(s\right)}_{s=j\omega }=M_{T}\left(A,\phi \right)C_{FOPD}\left(s\right)P_{c}{\left(s\right)|}_{s=j\omega }=-1,$$

so one can get the magnitude and the phase equations,

$$\begin{array}{cc} & |M_{T}\left(A,\phi \right)C_{FOPD}\left(s\right)P_{c}{\left(s\right)|}_{s=j\omega }=1,\\ & \arg{\left[M_{T}\left(A,\phi \right)C_{FOPD}\left(s\right)P_{c}\left(s\right)\right]}_{s=j\omega }=-\pi . \end{array}$$

Thus, all the ω satisfying (23) can be treated as the gain crossover frequencies $\omega_{gc}$ with A=1 and $\phi =\phi_{m}$ for the system (18) in Figure 5. Because equations (23) and (32) are equivalent, every frequency ω corresponding to the point on the curve of $K_{p}$ versus $K_{d}$ in parameter plane ($K_{p},K_{d}$) can also be treated as $\omega_{gc}$.

Thus, with the specified $\omega_{c}$, $\phi_{m}$, a fixed μ and a fixed $\omega_{o}$, the other two FOADRC parameters $K_{p}$ and $K_{d}$ can be determined at a point. Besides, the point should be tested whether it is in the stable region. Then, sweeping over the μ∈(0,2), a curve in the ($K_{p}$, $K_{d}$, μ)-space can be determined. All the points on this curve can be guaranteed to satisfy the two specifications $\omega_{c}$ and $\phi_{m}$. Then, Sweeping all the $\omega_{o}\in \left(\omega_{gc},\omega_{max}\right)$, one can obtain a three-dimensional graph about $K_{p}$, $K_{d}$ and μ. Every point on this graph corresponding to the parameters ($K_{p},K_{d},\mu$) of the FOADRC satisfying the pre-specified $\omega_{gc}$ and $\phi_{m}$.

**Figure 8:**
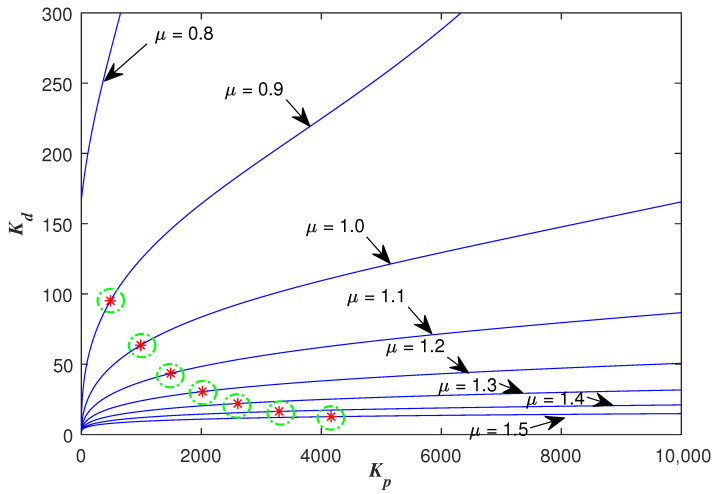
The designed $K_{p}$ and $K_{d}$ with sweeping all the optional μ∈(0,2).

**Figure 9:**
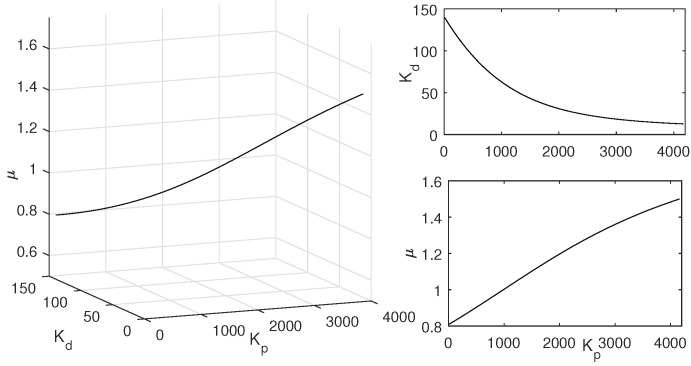
The designed $K_{p}$ and $K_{d}$ with all the μ∈(0,2).

**Figure 10:**
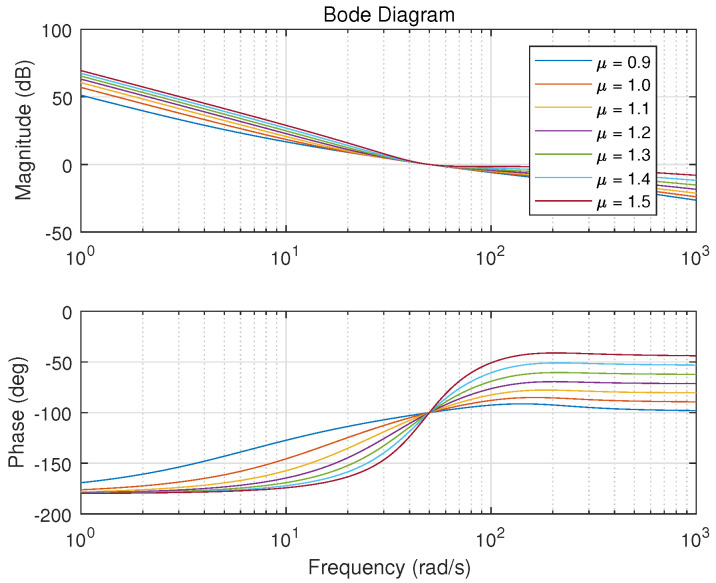
Bode plots with different μ.

**Figure 11:**
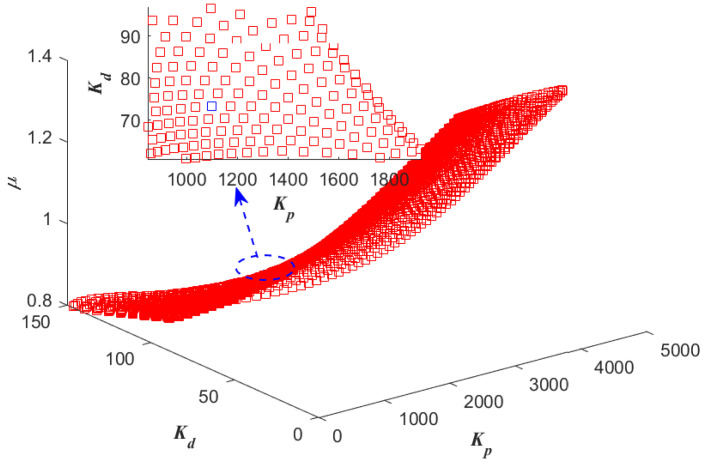
All the $K_{p}$, $K_{d}$, and μ with $\phi_{m}=80^{\circ }$ and $\omega_{gc}=50\;\mathrm{rad}/s$.

One example is given as: With the pre-specified $\phi_{m}=80^{\circ }$, $\omega_{gc}=50\;\mathrm{rad}/s$ and fixed μ=0.9, $\omega_{o}=200\;\mathrm{rad}/s$, the point corresponding to the parameters ($K_{p},K_{d}$) is determined as a red star ’A’ shown in Figure 6. Sweeping all the μ in (0,2), a series red stars can be obtained shown in Figure 8 and visualized in three-dimensional parameter space which is shown in Figure 9. Every point on this curve meets $\omega_{gc}=50\;\mathrm{rad}/s$ and $\phi_{m}=80^{\circ }$ according to Figure 10 with the $\omega_{o}=200\;\mathrm{rad}/s$, simultaneously. Sweeping all the $\omega_{o}$ in ($\omega_{gc}$, 800), one can obtain a three-dimensional scatter plot of $K_{p}-K_{d}-\mu$ as shown in Figure 11. Every point corresponding to the parameters satisfies the frequency-domain specifications $\omega_{gc}=50\;\mathrm{rad}/s$ and $\phi_{m}=80^{\circ }$.

#### 4.2.2. The Optimal FOADRC Satisfying Time-Domain Specifications

Given all the parameters of the FOADRC corresponding to the point on the above curve in Figure 9, the step and load-disturbance response simulation are implemented and the ITAE of the trial is calculated in MATLAB/Simulink. The ITAE is calculated using (33), which is the sampling form of (30)

(33) $$J_{ITAE}=\sum_{n=1}^{\frac{T}{\Delta t}}t\left[n\right]\left|e\left[n\right]\right|\Delta t.$$

**Figure 12:**
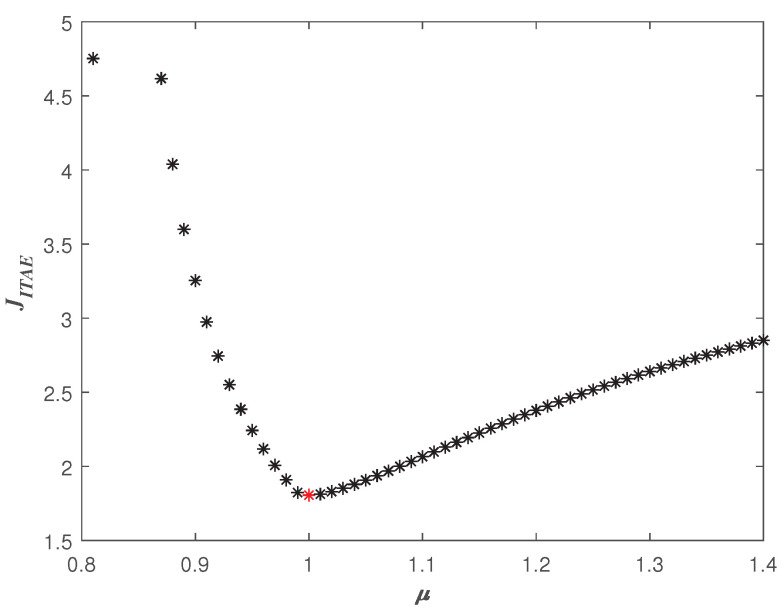
$J_{ITAE}$ with different μ.

**Figure 13:**
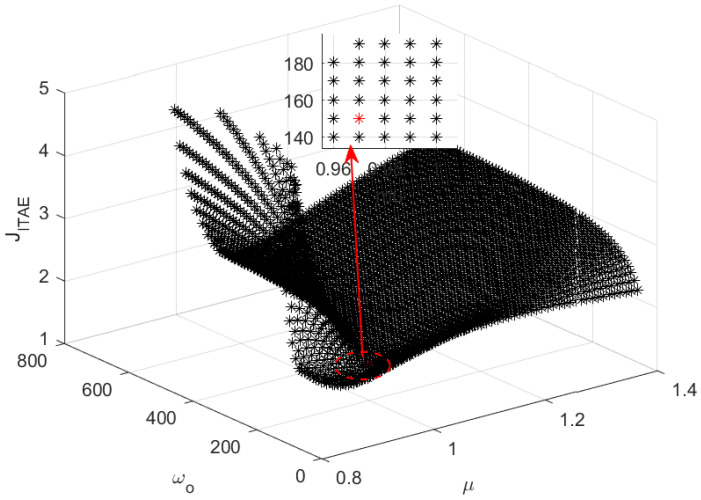
All the μ, $\omega_{o}$, and $J_{ITAE}$ with $\phi_{m}=80^{\circ }$ and $\omega_{gc}=50\;\mathrm{rad}/s$.

Thus, one can obtain the correspondence plot between μ and $J_{ITAE}$ as shown in Figure 12. And the smallest $J_{0}$ corresponding to the μ=1.0 is shown as a red star in Figure 12. Sweeping all ω the in ($\omega_{gc}$, 800), the three-dimensional scatter plot of $\mu -\omega_{o}-J_{ITAE}$ is shown in Figure 13. According to Figure 13, the smallest $J_{min}$ can be determined, and the parameters ($K_{p},K_{d},\omega_{o},\mu$) of the optimal FOADRC corresponding to the $J_{min}$ can be obtained.

**Figure 14:**
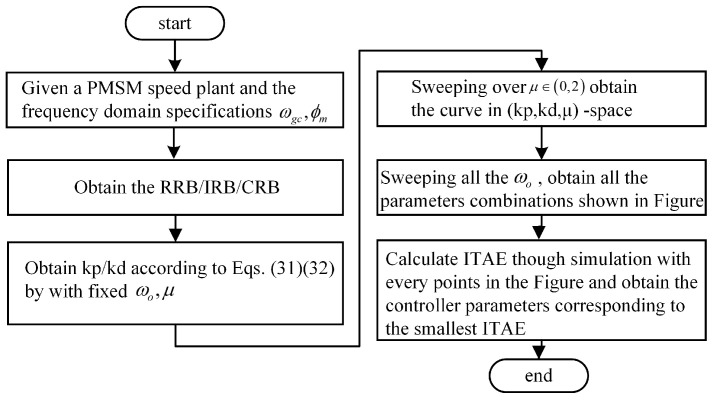
The design guidelines summary.

In summary, the design guidelines are shown in Figure 14.

### 4.3. IOADRC Design Strategy

The design strategy for IOADRC satisfying the user-specified frequency specifications $\omega_{gc}$ and PM is also given with a short explanation. For a fair comparison with FOADRC, set the same $\omega_{o}$ with FOADRC. Just as analyzed above, the parameters $K_{p}$ and $K_{d}$ with μ=1 can be determined according to Equations (26) and (27), with $\omega =\omega_{gc}$, $\phi =\phi_{m}$.

## 5. Simulation Results

In this section, simulation studies are carried out on PMSM speed servo system compared with IOADRC and typical PID controller [42]. The tuning strategy for typical PID controller is based on the frequency-domain specifications (gain crossover frequency, phase margin and flat phase constraint).

### 5.1. Tracking Performance

Given the frequency-domain specifications $\omega_{gc}=10\;\mathrm{rad}/s$ and $\phi_{m}=60^{\circ }$, the optimal FOADRC is obtained applying the above proposed method in Section 4,

(34) $$K_{p1}=123.59,K_{d1}=36.248,\mu =0.74,\omega_{o}=40\;\mathrm{rad}/s.$$

For fair comparison, the IOADRC is designed with the same frequency-domain specifications $\omega_{gc}=10\;\mathrm{rad}/s$ and $\phi_{m}=60^{\circ }$ and the same $\omega_{o}=40\;\mathrm{rad}/s$ according to the Section 4.3, so that one can get

(35) $$K_{p2}=202.703,K_{d2}=18.282.$$

Similarly, the typical PID controller can be designed with the same frequency-domain specifications as

(36) $$C_{PID}\left(s\right)=0.719+\frac{1.7416}{s}+0.006\;s.$$

**Figure 15:**
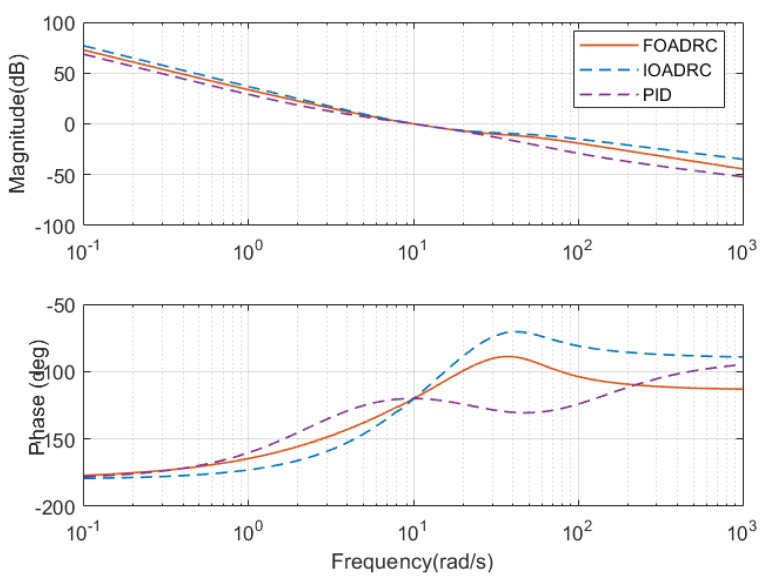
Open-loop Bode diagrams of three control systems.

**Figure 16:**
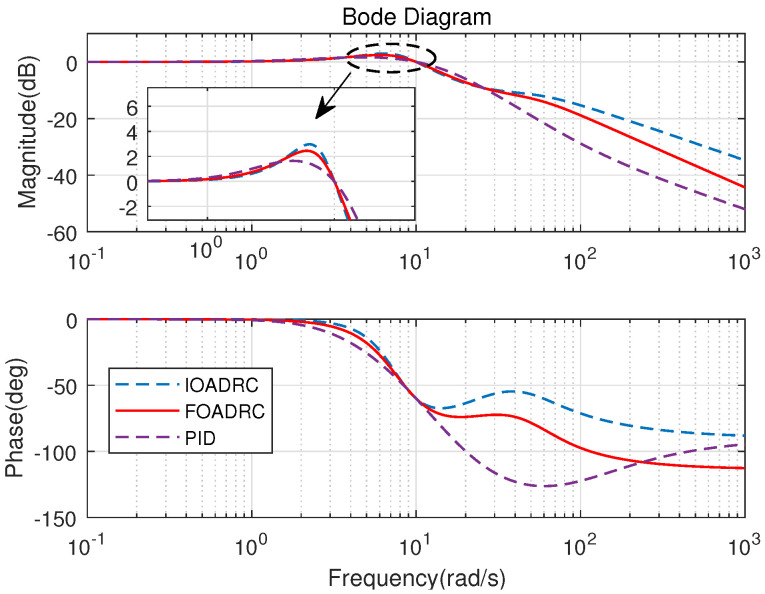
Closed-loop Bode diagrams of three control systems.

The open-loop Bode plots of the three control systems are presented in Figure 15. Three control systems have the same $\omega_{gc}$ and $\phi_{m}$. The closed-loop Bode plots of three control systems are shown in Figure 16. Figure 16 shows that the designed IOADRC control system has the biggest resonance peak, which means the IOADRC control system has the biggest overshoot than other control systems. The designed PID control system has the smallest overshoot with the smallest resonance.

**Figure 17:**
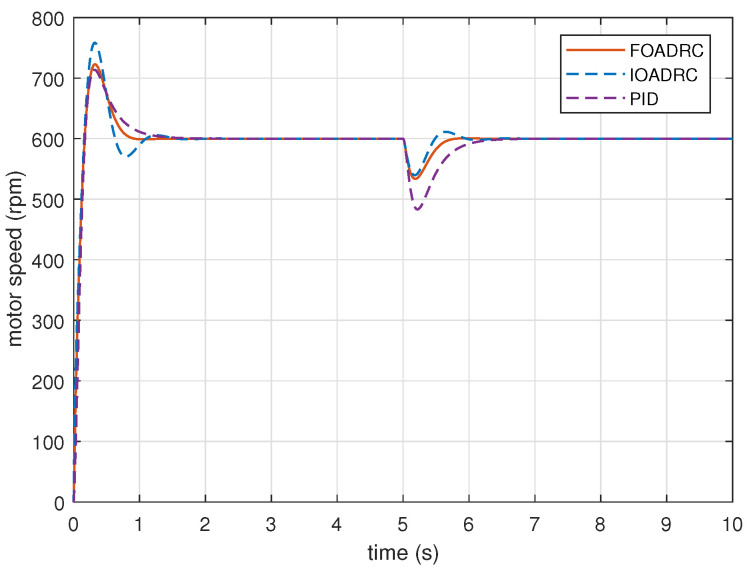
The speed and anti-load disturbance responses of three control systems (simulation).

**Figure 18:**
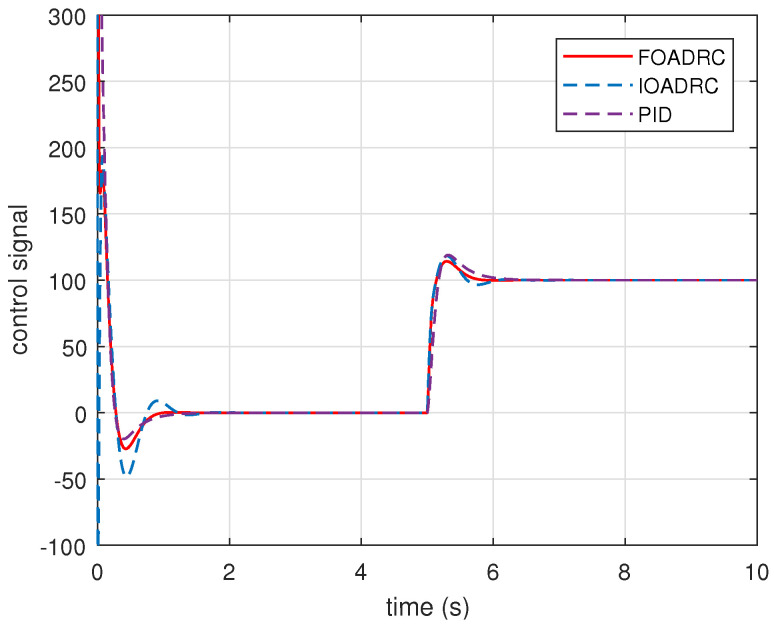
Control signals of three control systems.

Given the reference speed as 600 rpm, PMSM speed responses are performed, using the designed FOADRC, IOADRC, and PID controller. The simulation results are shown in Figure 17 and Figure 18. The comparison results are shown in Table 1.

Obviously, the designed PID control system has the smallest overshoot and the designed FOADRC control system has the biggest overshoot. The overshoots of three control systems are consistent with the analysis in Figure 16. However, comparing with the IOADRC control system, the designed FOADRC control system has shorter settling time (0.7369 s) and smaller overshoot (20.5%); comparing with PID control system, the designed FOADRC control system has a shorter settling time, although the overshoot is bigger. Overall, the designed FOADRC control system achieves the best tracking performance by comparison with $J_{ITAE}$ and can achieve a compromise between overshoot and settling time.

### 5.2. Robustness to External Disturbance

**Figure 19:**
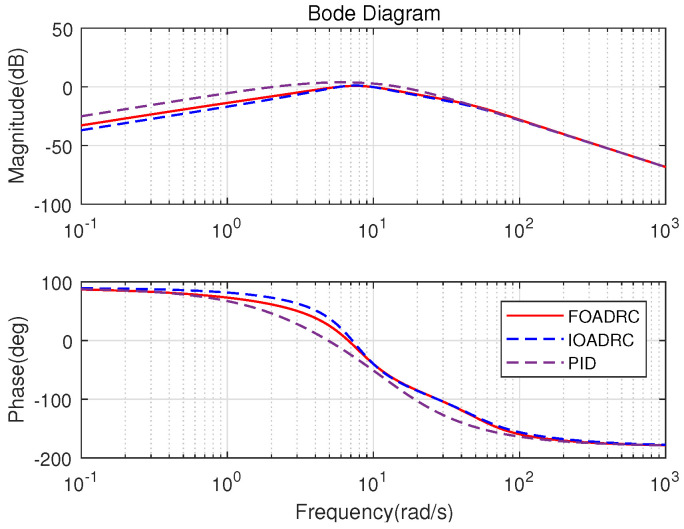
Sensitivity Bode diagrams of three control systems.

The process sensitivity Bode plots (P(s)/(1+C(s)P(s))) for three control systems are shown in Figure 19. Figure 19 shows that the designed FOADRC and IOADRC control systems have similar anti-load performance, and both outperform PID control system. Injecting the load disturbance when the motor speed is running, the anti-load disturbance responses of three control systems are performed as shown in Figure 17, and the comparison results are shown in Table 1.

As shown in Figure 19, the designed FOADRC and IOADRC control systems have the similar speed drop, and both are less than the speed drop of the PID control system. According to Figure 17 and Table 1, the designed PID control system has the biggest speed drop (19.47%) and recovery time; the speed drop shows that the anti-load disturbance performances of FOADRC and IOADRC control systems are basically the same and both significantly smaller than the PID control system, which also can be seen clearly from $J_{ITAE}$. These results are consistent with the analysis from Figure 19.

## 6. Experiment Results and Discussion

**Figure 20:**
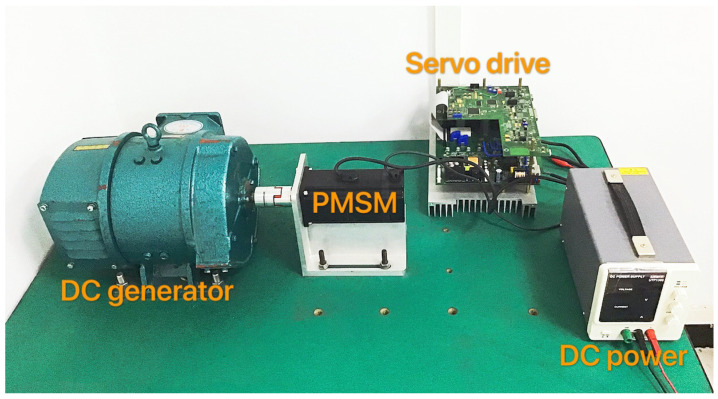
The PMSM speed control platform

**Figure 21:**
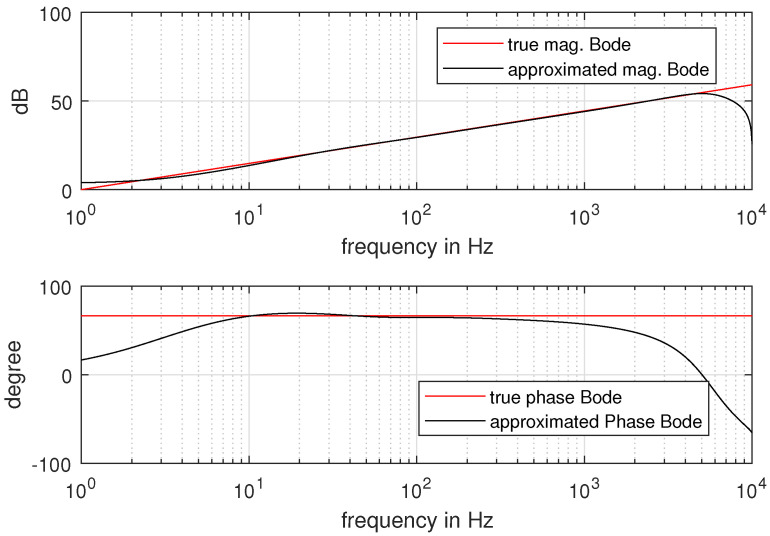
Comparison of approximated Bode plot and true Bode plot.

In this section, the real PMSM speed control experiments are carried out to verify the effectiveness of the proposed tuning strategy for FOADRC and IOADRC. The laboratory PMSM platform is shown in Figure 20. The platform consists of a PMSM, which the model is Sanyo-P50B08075HXS, a DC generator, a DC power, a servo driver, and a PC. The servo drive is based on the TMS320F28335 DSP and control algorithm implementation based on C-program. The sampling frequency of the current-loop is 8  kHz and the velocity-loop is 1.6 kHz. The fractional-order operator $s^{0.74}$ is implemented by the impulse-response-invariant-discretization (IRID) method [36] and has the following form (Equation (37)), with the sampling period $T_{s}=0.00625\;s$, and the comparison of approximated Bode plot and true Bode plot is shown in Figure 21.

(37) $$s^{0.74}\approx \frac{N}{D},$$

$$\begin{array}{cc} & N=z^{5}-3.27103z^{4}+4.00345z^{3}-2.22461z^{2}+0.529238z-0.0370365,\\ & D=0.003855z^{5}-0.009175z^{4}+0.007067z^{3}-0.001626z^{2}-0.0001659z+5.35515\times 10^{-5}. \end{array}$$

### 6.1. Tracking Performance

**Figure 22:**
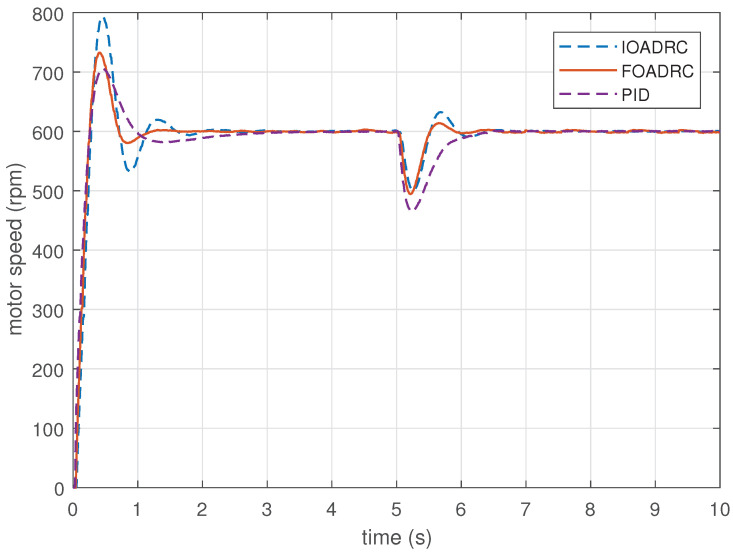
The speed and anti-load disturbance responses of three control systems (experiment).

Setting the reference speed as 600 rpm, PMSM speed step response experiments are performed, using the designed FOADRC, IOADRC, and PID controller. The experimental results are presented in Figure 22, and the comparison results are shown in Table 2.

Figure 22 shows that the designed PID control system has the smallest overshoot, and the designed IOADRC control system achieves the biggest overshoot, which are consistent with the analysis from Figure 16 and the simulation results in Figure 17. Comparing with the IOADRC control system, the designed FOADRC control system has shorter settling time (0.985 s) and smaller overshoot (22.1%); the designed PID control system has the smallest overshoot but the longest settling time. In summary, the FOADRC control system achieves the best tracking performance according to the $J_{ITAE}$ and can achieve a compromised performance in terms of overshoot and settling time between IOADRC and PID control systems.

### 6.2. Robustness to External Disturbance

Rejecting the same disturbance, the anti-load disturbance responses of three control systems are performed in Figure 22. As can be seen visually from Figure 22, IOADRC and FOADRC control systems achieve the similar speed drop and both less than the speed drop of the PID control system. There result are consistent with the analysis from Figure 19 and the simulation results in Figure 17. The designed PID control system has the biggest speed drop (19.47%) and recovery time. The IOADRC and FOADRC control systems have basically similar speed drop (16.6% and 17.6%), and the anti-load disturbance performance of IOADRC has a little oscillation. The detailed comparison results are shown in Table 2. In summary, the proposed FOADRC can achieve the best control performance in terms of anti-load over IOADRC and PID controller.

### 6.3. Discussion

Comparing with the traditional IOADRC, the proposed FOADRC can achieve better tracking performance with a smaller overshoot, and the value of the $J_{ITAE}$ is smaller. On the other hand, the proposed FOADRC has a little bigger speed drop when inputs load comparing with IOADRC. However, recover time is less than IOADRC and has a smaller value of $J_{ITAE}$. Comparing with the traditional PID controller, the proposed FOADRC can achieve better disturbance rejection performance with less speed drop when inputs load. On the other hand, the proposed FOADRC has a bigger overshoot comparing with PID controller. However, the settling time of the proposed FOADRC is less than the one of PID controller and has less $J_{ITAE}$.

## 7. Conclusions

The paper proposes a FOADRC with a FOPD controller for the PMSM speed servo system, which is identified through a nonlinear identification method. A stabilization method is presented to obtain the stabilizing FOPD controller for the PMSM speed servo plant via D-decomposition. Furthermore, a synthesis is proposed for the FOADRC design to meet user-specified frequency-domain specifications, i.e., phase margin and gain crossover frequency, and the time-domain specification, i.e., ITAE, simultaneously. The design method is also applicable to IOADRC. The designed optimal FOADRC/IOADRC can get desired control performance as satisfying two given frequency-domain specifications and the optimal $J_{ITAE}$. The simulation and experiment are carried out for the PMSM speed servo, comprised of the traditional IOADRC and PID controller. Based on the simulation and experiment results, the proposed FOADRC can achieve better tracking performance and more robustness to external disturbance. The $J_{ITAE}$ for speed tracking of the designed FOADRC are 52.59% and 55.36% better than the $J_{ITAE}$ of IOADRC and PID controller, respectively. Obviously, the settling time of the designed FOADRC is the shortest (0.7369 s), which is 32.99% and 49.22% less than the settling time of the designed IOADRC and PID controller. Besides, the $J_{ITAE}$ for anti-load disturbance of the designed FOADRC is 17.11% and 52.50% better than the $J_{ITAE}$ of IOADRC and PID controller, respectively.

## Figures and Tables

**Table 1:** Comparison results of three control systems (simulation).

	Speed Tracking			Anti-Load Disturbance	
Controller	Overshoot (%)	Settling Time (s)	$J_{\mathrm{ITAE}}$	Speed Drop (%)	$J_{\mathrm{ITAE}}$
FOADRC	20.5	0.7369	19.2	12.07	110.32
IOADRC	26.4	0.9831	27.18	11.08	124.3
PID	19	0.985	28.05	19.47	301.45

**Table 2:** Comparison results of three control systems (experiment).

	Speed Tracking			Anti-Load Disturbance	
Controller	Overshoot (%)	Settling Time (s)	$J_{\mathrm{ITAE}}$	Speed Drop (%)	$J_{\mathrm{ITAE}}$
FOADRC	22.1	0.985	28.72	17.6	174.814
IOADRC	32.3	1.47	60.58	16.6	210.8931
PID	19	1.94	64.3414	19.47	368.0378

## Data Availability

Date sharing not applicable.

## Abbreviations

FOADRC	fractional-order active disturbance rejection control
FOPD	fractional-order proportional-derivative
ESO	extended state observer
PMSM	permanent magnet synchronous motor
ADRC	active disturbance rejection control
IOADRC	integer-order active disturbance rejection control
PID	proportional-integral-derivative
FO	fractional-order
FOPI	fractional-order proportional-integral
FO(PD)	fractional-order (proportional-derivative)
FOPID	fractional-order proportional-integral-derivative
ISE	integral square error
ITAE	integral time absolute error
LADRC	linear active disturbance rejection control
GADRC	generalized active disturbance rejection control
GA	genetic algorithm
FADRC	fractional active disturbance rejection control
FOESO	fractional-order extended state observer
FOS	fractional order system
PI	proportional-integral
RRB	real root boundary
IRB	infinite root boundary
CRB	complex root boundary
PM	phase margin
IRID	impulse-response-invariant-discretization
